# Operando DRIFT-MS for studying the oxidative steam reforming of ethanol (OSRE) reaction

**DOI:** 10.1016/j.mex.2023.102169

**Published:** 2023-04-07

**Authors:** César Rodríguez, Sonia Moreno, Rafael Molina

**Affiliations:** Estado Sólido y Catálisis Ambiental, Departamento de Química, Facultad de Ciencias, Universidad Nacional de Colombia, AK 30 No. 45-03, Ciudad Universitaria, Bogotá D.C., Colombia

**Keywords:** Operando DRIFT-MS, OSRE, Hydrogen, Operando experimental design, Particle size, Operando Diffuse Reflectance Infrared Fourier Transform Spectroscopy coupled with Mass Spectrometry (DRIFT-MS) for studying the Oxidative Steam Reforming of Ethanol (OSRE) reaction

## Abstract

An operando DRIFT-MS system (Diffuse Reflectance Infrared Fourier Transform Spectroscopy coupled with Mass Spectrometry) was designed and set up to study the oxidative steam reforming of ethanol reaction (OSRE). This reaction involves the mixture of water, ethanol and oxygen to produce mainly hydrogen, which is a rather attractive energy carrier. Spectroscopic monitoring of the process is a key tool to contribute to the understanding of: i) the dynamics on the catalyst surface, ii) the reaction mechanism and iii) the effect of the solid's properties on the catalytic process. In this sense, this document sets forth the experimental design that allows to carry out the study under operando DRIFT-MS conditions through time for the OSRE reaction. Selection criteria for parameters, materials, configuration, and experimental conditions are included, particularly optimizing the parameters of particle size and the dilution factor with KBr as well as the temperature and flow conditions for carrying out the reaction.

• Clear signals of the adsorbed species in IR that do not present interference by water in the reaction atmosphere.
• Simple assembly and online product detection by MS that allow to follow the change in the products of the OSRE reaction according to the temperature.
• Controlled entry of gases and quantification by loop injection.

## Graphical abstract

**Figure:**
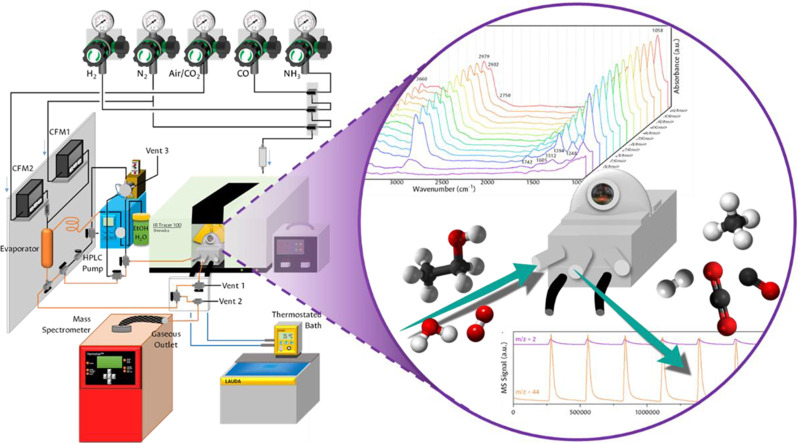

Specifications table

**Table:** 

Subject area:	Chemistry

More specific subject area:	*Catalysis*
Name of your method:	*Operando Diffuse Reflectance Infrared Fourier Transform Spectroscopy coupled with Mass Spectrometry (DRIFT-MS) for studying the Oxidative Steam Reforming of Ethanol (OSRE) reaction*
Name and reference of original method:	*Not applicable*
Resource availability:	*Not applicable*

## Method details

### Background information

In 2002 the term operando was coined [1], referring to the simultaneous measurement of surface changes of heterogeneous catalysts by means of spectroscopic techniques (*in situ* analysis) and online monitoring of reaction products by mass spectrometry or chromatography [2]. This approach to the study of catalytic processes facilitates the understanding of surface phenomena and dynamics on a deeper level, offering alternatives, amongst other options, in order to reveal the missing information in the approximation to the different reaction mechanisms under real process conditions by following their reaction products, this simultaneous monitoring makes the data obtained in the operando processes closer to reality than just having an *in situ* study [1], [2], [3], [4]. The scenario described obviously requires a rigorous experimental design accompanied by the utmost and best combination of spectroscopic techniques such as infrared (IR), Raman and ultraviolet-visible (UV–Vis) as well as Nuclear Magnetic Resonance (NMR).

**Fig. 1:**
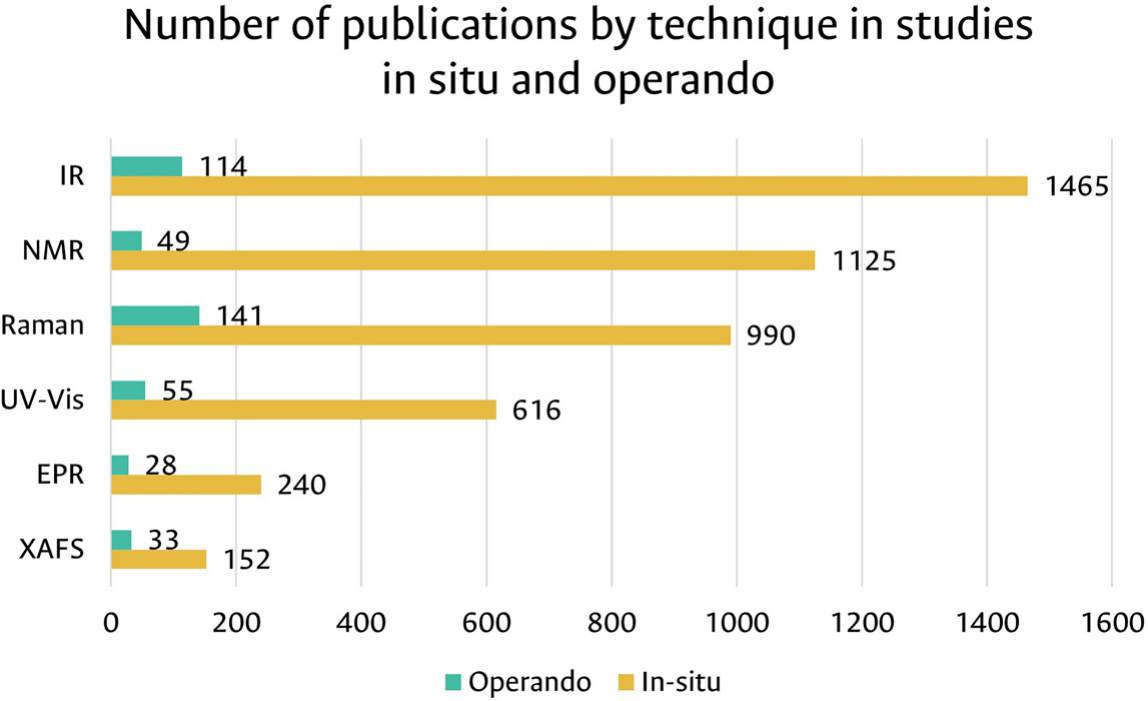
Scopus (October 2022) “Catalysis” AND “in situ/operando” AND “Technique”.

As illustrated in Fig. 1, a significant number of studies reports in-situ measurements of catalysts with these techniques, but few record operando related studies. This could be attributed to the complex couplings required by many of the techniques with online analysis systems of the liquid or gaseous currents generated in the catalytic reactions, due to the large void volumes or heat and mass transfer gradients [2].

IR spectroscopy is one of the most useful techniques for the characterization of catalysts [5]. However, beyond this general application and thanks to the vibration of the molecules due to the absorption of the irradiated photons (vibrational energy), IR allows for the identification and monitoring of the species adsorbed on the surface of the catalyst, observing the molecular changes and determining the dispersion of active agents in a support as well as regards surface activity [5,6]. This technique comprises two main analysis configurations which are usually used, transmittance spectroscopy (FTIR) and diffuse reflectance spectroscopy (DRIFT).

The DRIFT configuration is the one most widely used in heterogeneous catalysis [3,^4^,[7], [8], [9], [10], [11], [12] since opaque solids are usually studied, mainly oxides, which present low absorption coefficients of infrared radiation that could not be easily used in the transmittance mode, therefore, their high refractive index is used to obtain information on the solid. Additionally, powdered materials can be studied (in their original state without modifying the properties of the solid [13]) since they do not need to be compressed to form a tablet and the packing or accommodation in the measurement accessories simulates a catalytic bed quite well [14,15]. Through the admission of different controlled atmospheres and the use of temperature programs, it is possible to detect, with high sensitivity, the surface species (compared to a configuration in FTIR) and emulate the reaction conditions in order to obtain information under real working conditions of the catalyst [2,^3^,10].

As for the couplings most used to monitor the products in DRIFT experiments, mass spectrometry (MS) has been employed, which allows the instantaneous detection of the different reaction products based on time, facilitating the identification of products in low quantities, sporadic formations and intermediaries [2,^3^,^6^,^7^,^10^,^16^,17]. In addition, this system registers, with high sensitivity, the instantaneous change (microseconds) in composition over time [18], supplying (and complementing at the same time) the limitations of the follow-up to the reaction when it is carried out by means of chromatography, where information is lost due to the dead time between injections.

With this incentive, the experimental set-up was designed to carry out the operando measurements, using infrared spectroscopy with Fourier transform in diffuse reflectance mode (DRIFT) in order to observe the surface changes of the organic species coupled to mass spectrometry (MS) equipment to monitor the oxidative steam reforming of ethanol (OSRE) process [11,[19], [20], [21], [22], [23], [24]. This reaction is quite attractive for renewable hydrogen production today and is one of the prominent ways to contribute to the energy transition with cleaner processes. To our knowledge, the OSRE reaction has not yet been reported under operando DRIFT-MS conditions.

To evaluate the set-up and protocols of the experimental design, firstly, an exhaustive review of the literature related to in-situ DRIFT configurations with molecules such as H_2_O and EtOH was carried out. It was established that the study of ethanol adsorption by DRIFT in-situ was initially evaluated in simple metallic systems of Pd, Pt and Rh [25], [26], [27] and, later, advanced to more complex systems such as Pt/TiO_2_ for the production of H_2_ by photo-catalytic means [28], Co/ZnO, Ni/Al_2_O_3_ and Ni/MgAl_2_O_4_ by steam reforming of ethanol [29], [30], [31], Cu/Nb_2_O_5_ by reforming of ethanol with steam and partial oxidation [32], Pt/CeZrO_2_ by steam reforming of ethanol and oxidative steam reforming of ethanol [33], and Ir/CeO_2_ by oxidative steam reforming of  ethanol [34].

On the other hand, the water-gas shift (WGS) reaction using Eu doped with ZrO_2_ as catalyst has also been reported [35] evidencing that the adsorption of water and ethanol in the in-situ reaction chamber by DRIFT is possible by means of a heating system (evaporator) and the use of an HPLC pump as feed or by cooling the liquid to 0 °C and bubbling with carrier gas in the reaction chamber.

In the described context, the materials and methods for the characterization of the oxidative steam reforming of ethanol (OSRE) reaction under operando conditions are described below.

## Materials and methods

### Assembly and fine-tuning of the reaction system

**Fig. 2:**
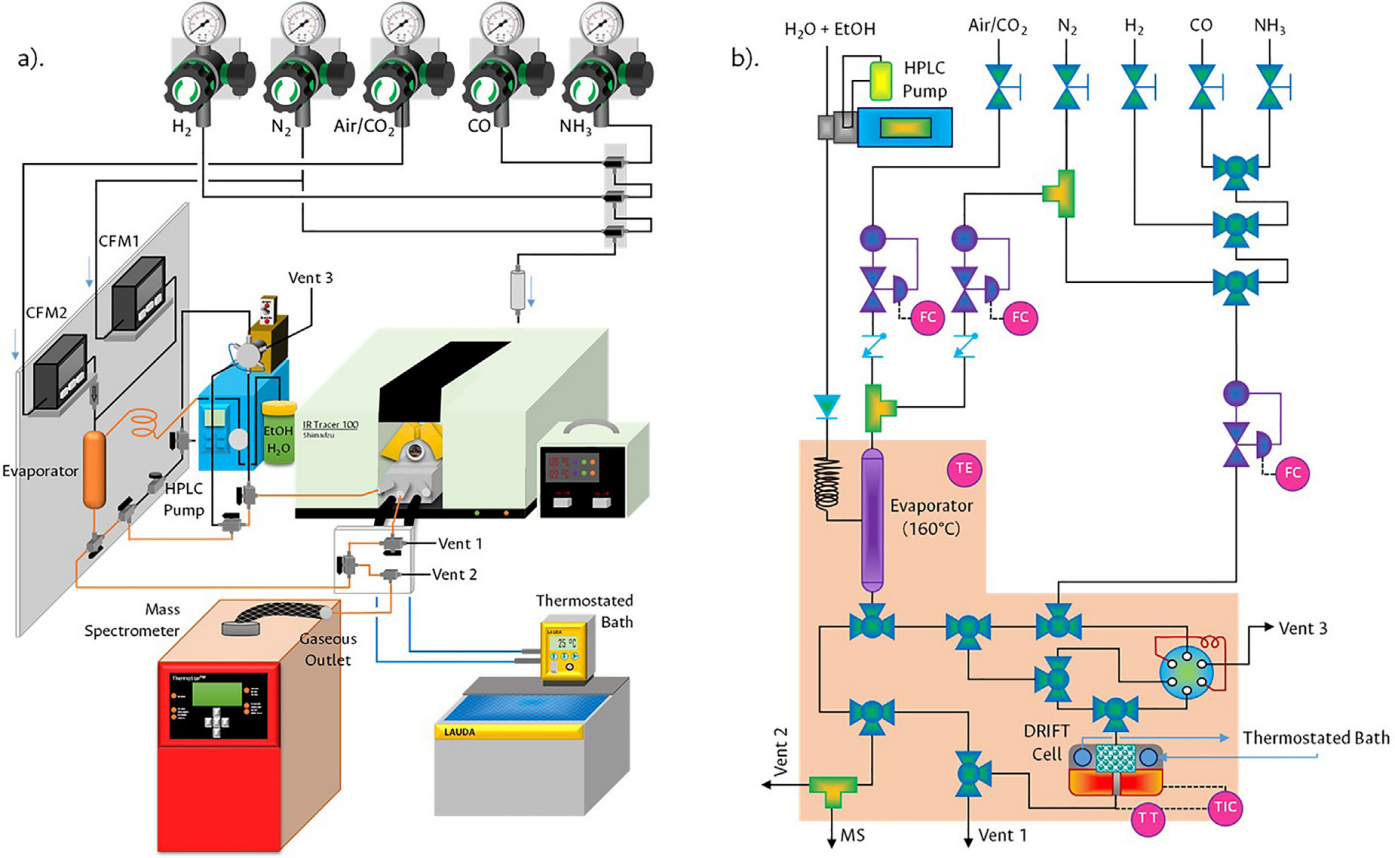
a) Setup design for DRIFT-MS experiments and b) P&ID instrumentation diagram.

Fig. 2(a) illustrates the design applied to carry out the OSRE reaction under operando conditions using online DRIFT-MS equipment, while Fig. 2(b) allows identifying the details of the general scheme of the reaction pathways.

**Fig. 3:**
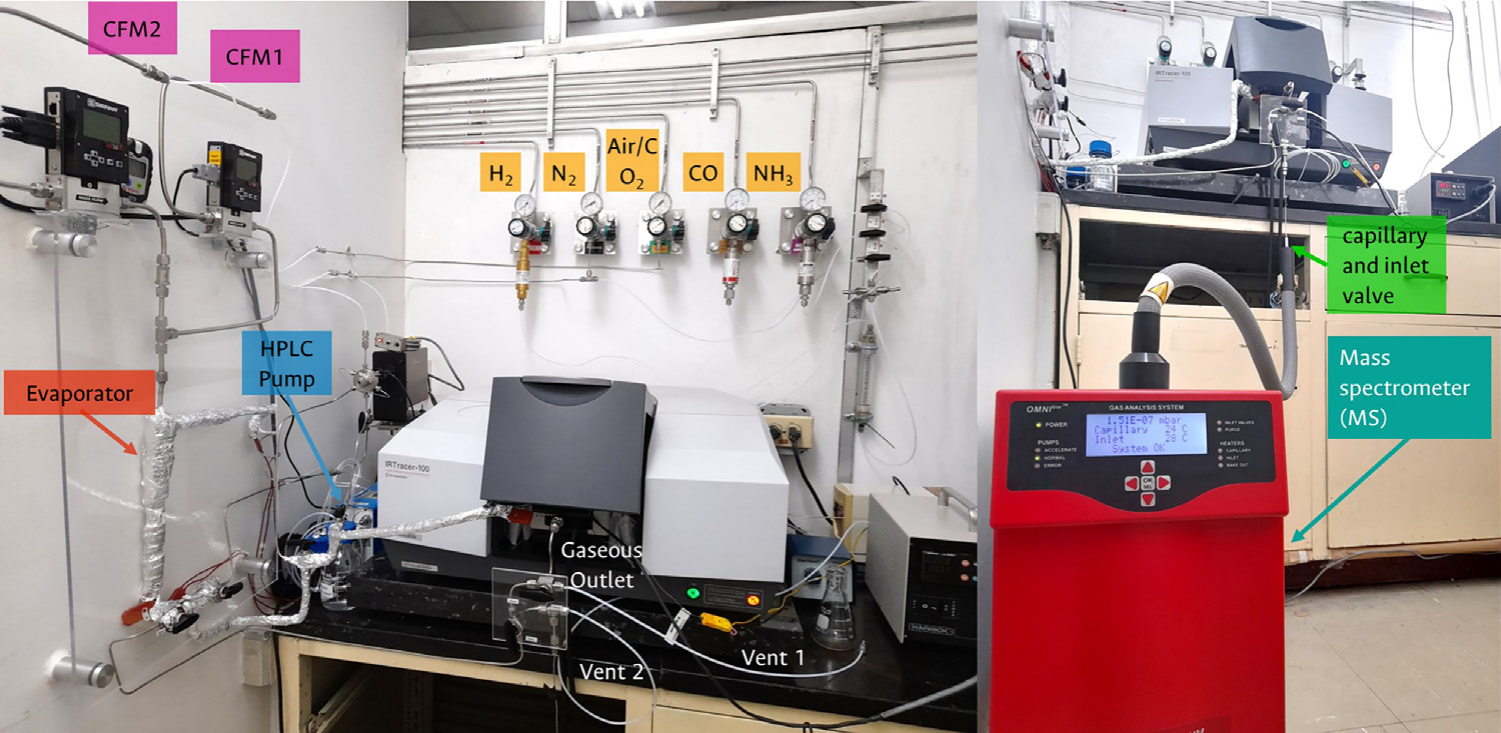
System for operando measurements using diffuse reflectance infrared spectroscopy coupled to mass spectrometry (DRIFT-MS).

The overall system consists of three main parts: (i) gas and liquid feed, (ii) reaction chamber arranged in the IR, and (iii) MS detection. Fig. 3 illustrates the complete experimental infrastructure, which includes the following equipment and supplies:

• A MasterFlex digital Piston Cole-Parmer HPLC pump with a maximum flow of 10.00 mL·min^-1^.
• Wisetherm HM-C10A rheostat and 300 W 220 V flexible silicone heater strip belts for feed lines.
• Two Sierra Instruments – Series 100 Smart Track model C100L mass flow controllers.
• A Shimadzu IRTracer-100 infrared (IR) spectrometer equipped with a standard infrared source (9600–50 cm^-1^), with a Ge/KBr beam splitter (7400–350 cm^-1^), an encapsulated KBr optic and a standard DLATGS detector (7800–350 cm^-1^). The spectroscopic cell for reaction is a Harrick chamber of high pressure (50 bar) and temperature (which is regulated from room temperature to 700 °C), with gas stream inlet/outlet, two ZnSe windows and one KBr window inside a Diffuse reflection Praying Mantis accessory.
• A Pfeiffer Vacuum OmniStar mass spectrometer (MS) equipped with a quartz capillary (T_Máx_=350 °C) for gas ingress, two tungsten filaments for electronic ionization (15–100 eV), an MVP 020 vacuum pump (*P* = 1×10^–8^ mbar), a quadrupole analyser (QMA 200 M) and two SEM/Faraday detectors.
• 1/8″ and 1/4″ Swagelok-type stainless steel tubing and connections, and an acrylic support specifically adapted for this set-up.
• An ANSI 304 SCH 10 stainless steel evaporator with an internal diameter of 2 cm, free height 12 cm with three connections without caps OD-OD for 1/4″ tubing.
• 6-way VICI valve with a Loop for 50 µL gases.
• Workstations for the entry of gases such as H_2_, N_2_, Dry air, CO_2_, CO and NH_3_.

### Praying mantis accessory settings

**Fig. 4:**
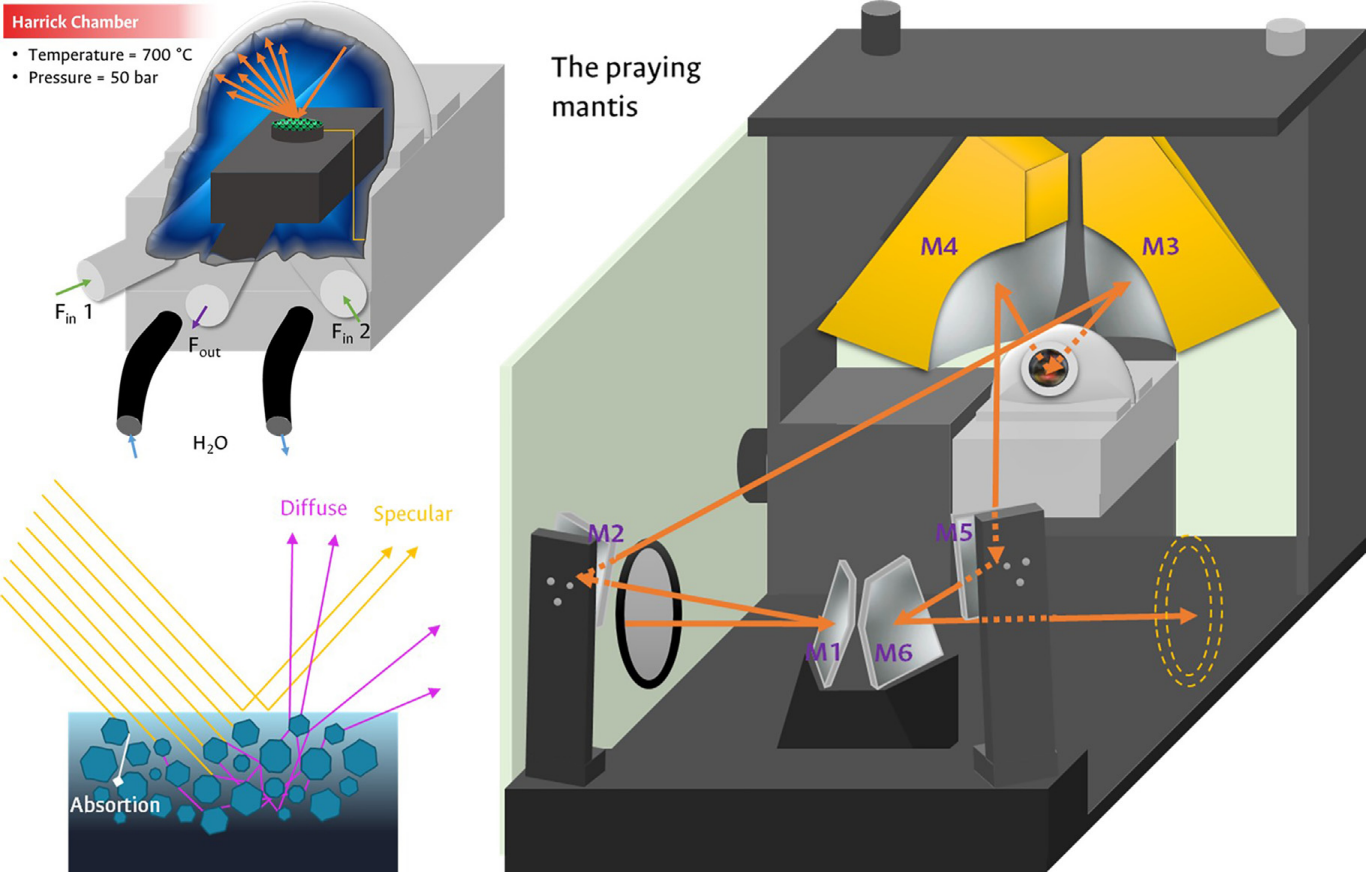
Types of surface reflections and Harrick camera with Praying Mantis accessory for experiments in DRIFT mode.

Light reflection phenomena allow us to identify three types of reflectance that can occur on the surface of a solid: specular reflection, diffuse reflection and absorption/reflection [15]. This requires an adjustment of the signals of the reaction chamber utilized. In Fig. 4 the types of reflectance are exemplified. The specular type corresponds to the radiation that does not have a change in its angle of incidence with that of exit, generally presented on smooth and uniform surfaces, while the diffuse type is registered on rough surfaces that alter the incident radiation by reflecting it in different directions; In addition to these two processes, part of the radiation can be absorbed and reflected in different directions [15]. Most solids present a mixture of these types of reflections in different proportions, depending on the composition and properties of the solid.

These reflections can be studied in a Harrick chamber arranged inside a Praying Mantis accessory, which correspond to very popular couplings in operando experiments for the measurement of physicochemical processes involving gas-solid interfaces [3,^4^,[8], [9], [10], [11],^33^,36]. This configuration allows operating in real reaction conditions on powdered catalysts, employing diffuse reflection to observe the changes on the surface of the molecules in different atmospheres and at certain temperatures [15]. Using diffuse reflection throughout a significant volume of the solid results in an increase in the vibration signals of the molecules on the surface over the specular reflection signals of the solid. For this reason, the specular contribution must be minimized and, simultaneously, the diffuse contribution must be maximized. This is achieved by attaching the Praying Mantis accessory to the analysis compartment of the Shimadzu IRTracer-100 and adjusting the position of the mirrors, using the adjustment device as a reference.

**Fig. 5:**
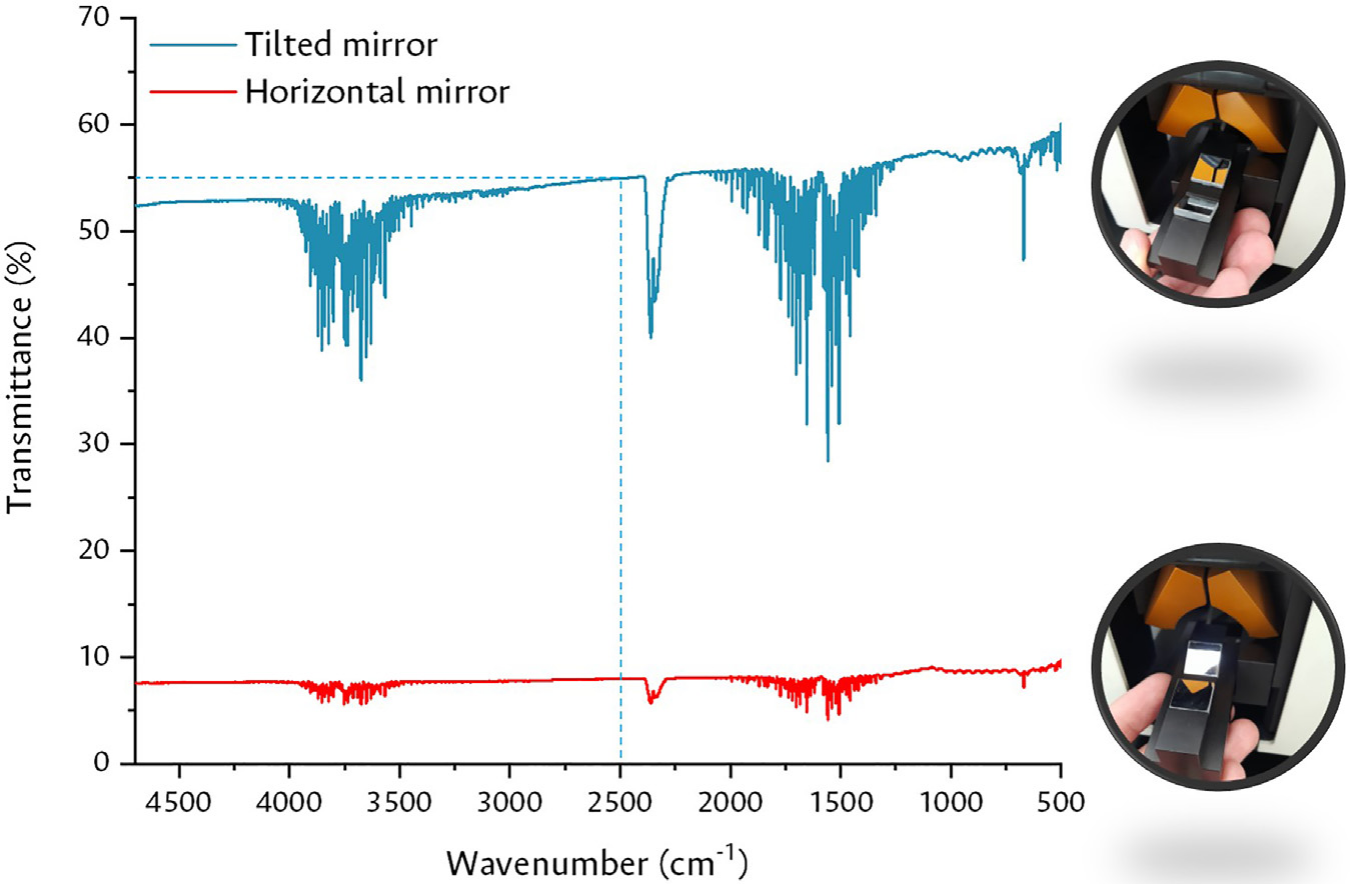
Diffuse (Tilted Mirror) and Specular (Horizontal Mirror) reflection adjustment.

Fig. 5 illustrates the result of the alignment of the mirror system, with a contribution of 55% of the diffuse radiation and 9% of the specular at 2500 cm^-1^. Once the adjustment was made, preliminary experiments were carried out to verify the status of the signals obtained.

### Preliminary experiments

An HPLC pump was used to continuously feed the H_2_O/Ethanol mixture to a preheated coil at 120 °C and, in parallel, a gaseous stream of dry air (as source of O_2_) and N_2_ (as carrier gas and internal standard) was added by independent mass flow controllers (Fig. 2b). These components were mixed in an evaporator maintaining the temperature at 160 °C throughout the process, ensuring that all components remain in the gas phase. The molar ratio used for the mixture was H_2_O/C_2_H_5_OH/O_2_/N_2_ = 3:1:0.5:5.

The evaporator outlet guides the gas stream through the valve system that enters the Harrick chamber and passes through the catalytic bed, arranged with a mixed oxide of Ni, Co, Mg and Al (which has been studied in the OSRE reaction) maintaining the system at 100 °C to avoid condensation processes. The output current of the Harrick chamber is transported to a “t” connection that allows the coupling between a vent and the MS inlet capillary, facilitating the entry of gases without generating a pressure difference due to the change in diameter in the tubing.

The experimental design began with tests of the ethanol adsorption signals by varying the particle size of the solid, considered a critical parameter for measurements in-situ and operando processes. For this, after a general sweep, three particle sizes were selected: 125, 53 and 45 µm, using ASTM 120, 270 and 325 sieves, respectively.

**Fig. 6:**
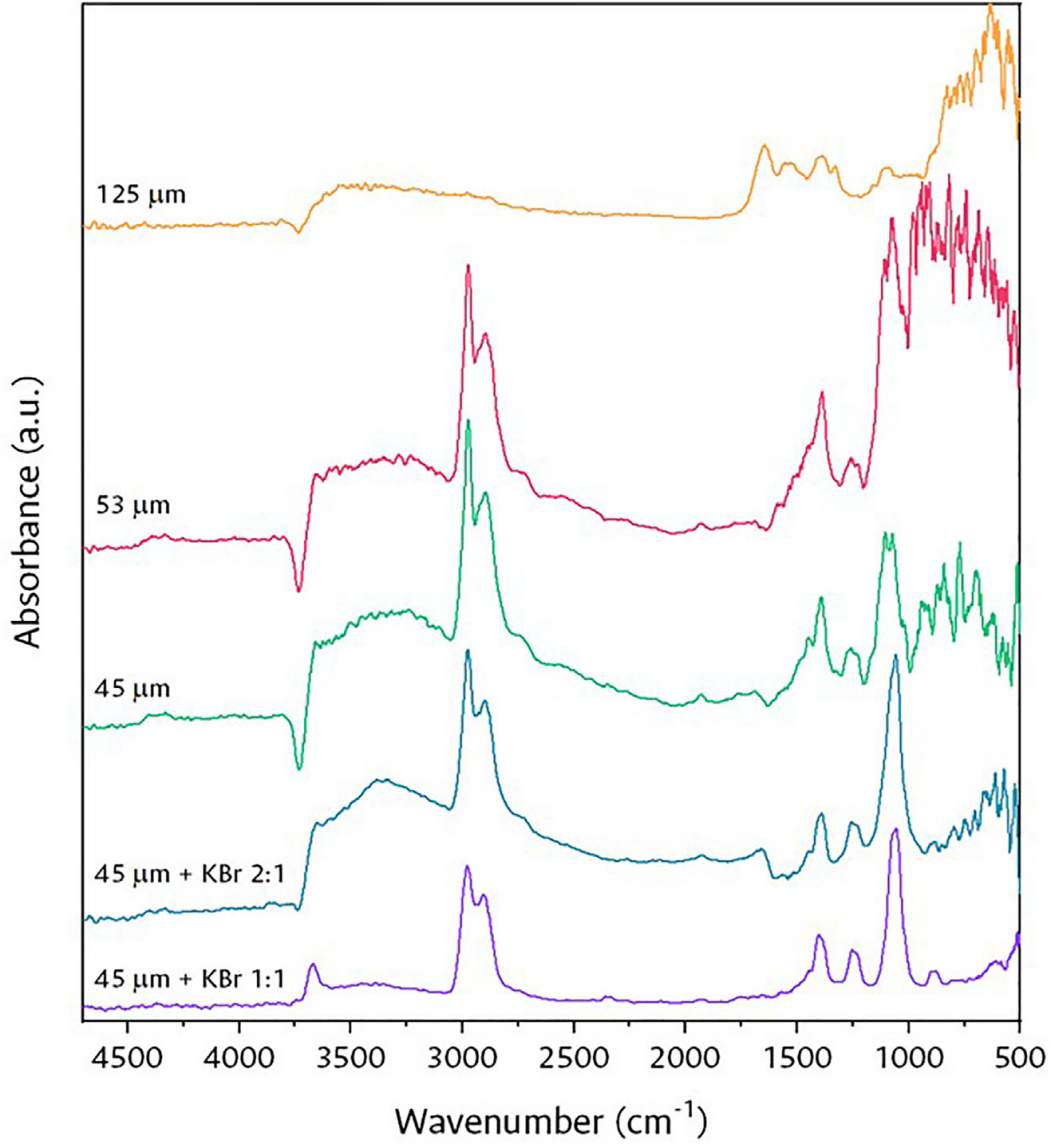
Effect of particle size and KBr dilution on signal resolution.

As can be seen in Fig. 6, at a smaller particle size, the signals of the adsorbed ethanol species are clearer and more intense, this is because large particles generally absorb too much IR radiation, thus, by decreasing the particle size, the total reflectance of the sample increases [14]. However, these still show a high contribution by specular reflection. This inconvenience was minimized by diluting a 45 µm fraction with KBr (a material that transmits radiation from 40,000 to 400 cm^-1^), thus improving the depth of radiation penetration thus improving the depth of radiation penetration when performing this mechanical mixture using an agate mortar and pestle [14]. The catalyst/KBr weight ratios used were 2:1 and 1:1, revealing that the greater amount of diluent considerably improves the spectra, recording “cleaner” signals, particularly at wavenumbers less than 1000 cm^-1^.

In summary, it was established that for a reasonably adequate signal, it is necessary to work with a particle size of less than 45 µm and to dilute the catalyst in KBr with a weight ratio of 1:1. Lower diluent ratios could be used to decrease interference but would also affect space velocity in the process.

## Experimental protocol to carry out the catalytic tests

### Catalyst activation

The reactor was packed with approximately 60 mg of mixture (catalyst/KBr = 1:1), leaving the surface smooth with the spade-type attachment. After adjusting the dome, a current of pure H_2_ was allowed to pass at a flow rate of 30 mL·min^-1^. Hydrogen is used to reduce the catalyst, since reforming reactions require a metallic phase to carry out the reaction [37]. The temperature was raised with a 10 °C·min^-1^ ramp to 600 °C and maintained for 1 h. Once this process was finished, the flow of the reducing gas was closed, and it was cleaned with N_2_ (30 mL·min^-1^) at the same temperature for 15 min. The reaction chamber was allowed to cool, and the spectrum of the reduced solid was taken as Background for the following steps.

### Ethanol adsorption

The reaction lines were heated to a temperature of 120 °C and introduced the feed streams of the HPLC pump (0.01 mL·min^-1^), air (5.8 mL·min^-1^) and N_2_ (7.7 mL·min^-1^) through the mass flow controllers to achieve a mixture in the evaporator with the indicated molar ratios. This gas stream passed through the reaction chamber at 100 °C (to avoid any type of condensation) and the initial amounts were detected by the signals in the MS. The data acquisition software used was LabSolutions IR Launcher, acquiring 80 scans with a resolution of 2 cm^-1^ in absorbance.

**Fig. 7:**
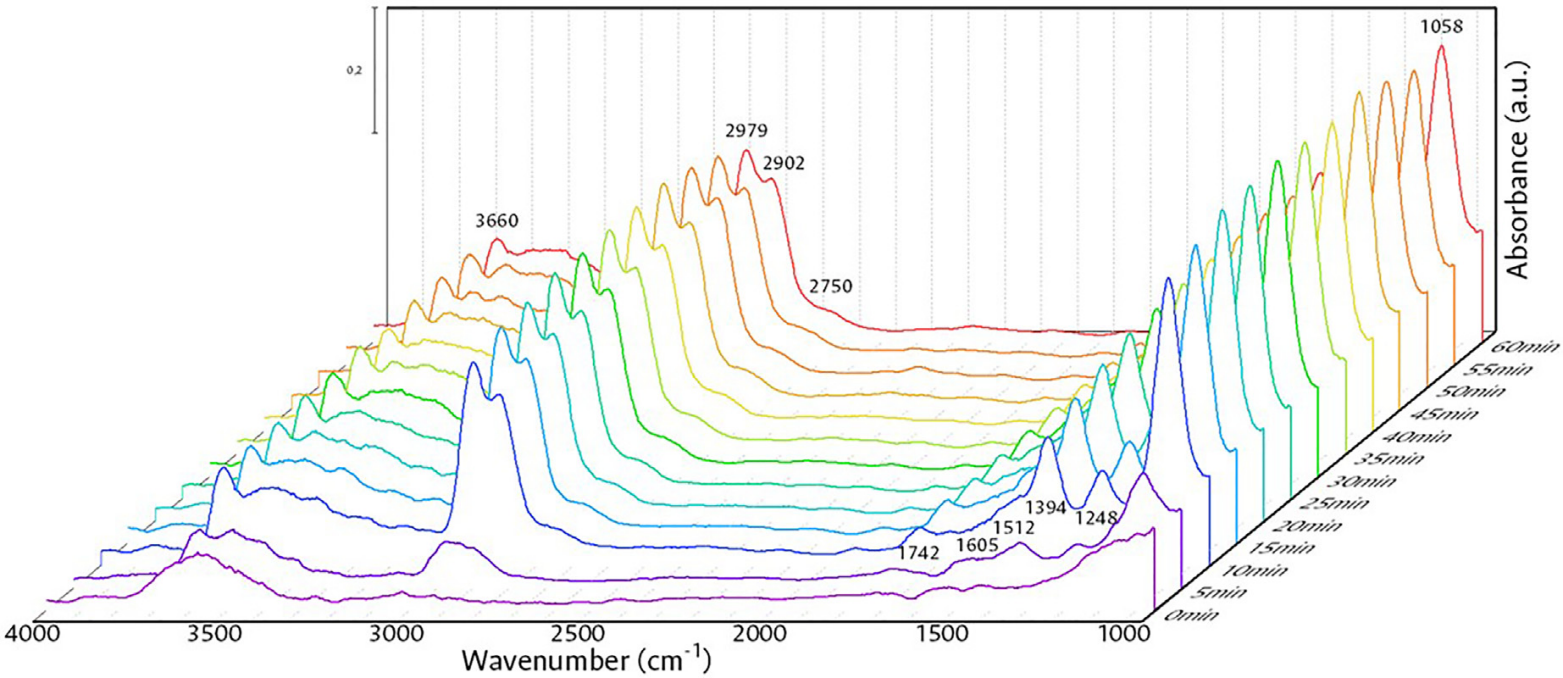
Ethanol adsorption at 100 °C.

By monitoring the adsorption of ethanol (Fig. 7) for 1 h at 100 °C, a maximum adsorption is found after 15 min and observing mainly the formation of Type II ethoxy species thanks to the chemisorption of ethanol, with a vibration ν(CO) at 1058 cm^-1^, ν_as_(CH_3_) and ν_as_(CH_2_) at 2979 and 2902 cm^-1^ respectively  and flexions δ_as_CH_3_ and δ_as_CH_3_ at 1394 and 1248 respectively, in addition to a vibration of the (OH) bond at 3660 cm^-1^ by Type II species due to the breaking of the O—H bond of ethanol [17,^33^,^36^,38].

The adsorption experiment demonstrates the effectiveness of the setup by feeding a constant amount of the mixture and allowing the monitoring of the species formed in the reaction chamber over time.

### Effect of temperature and evolution of species

**Fig. 8:**
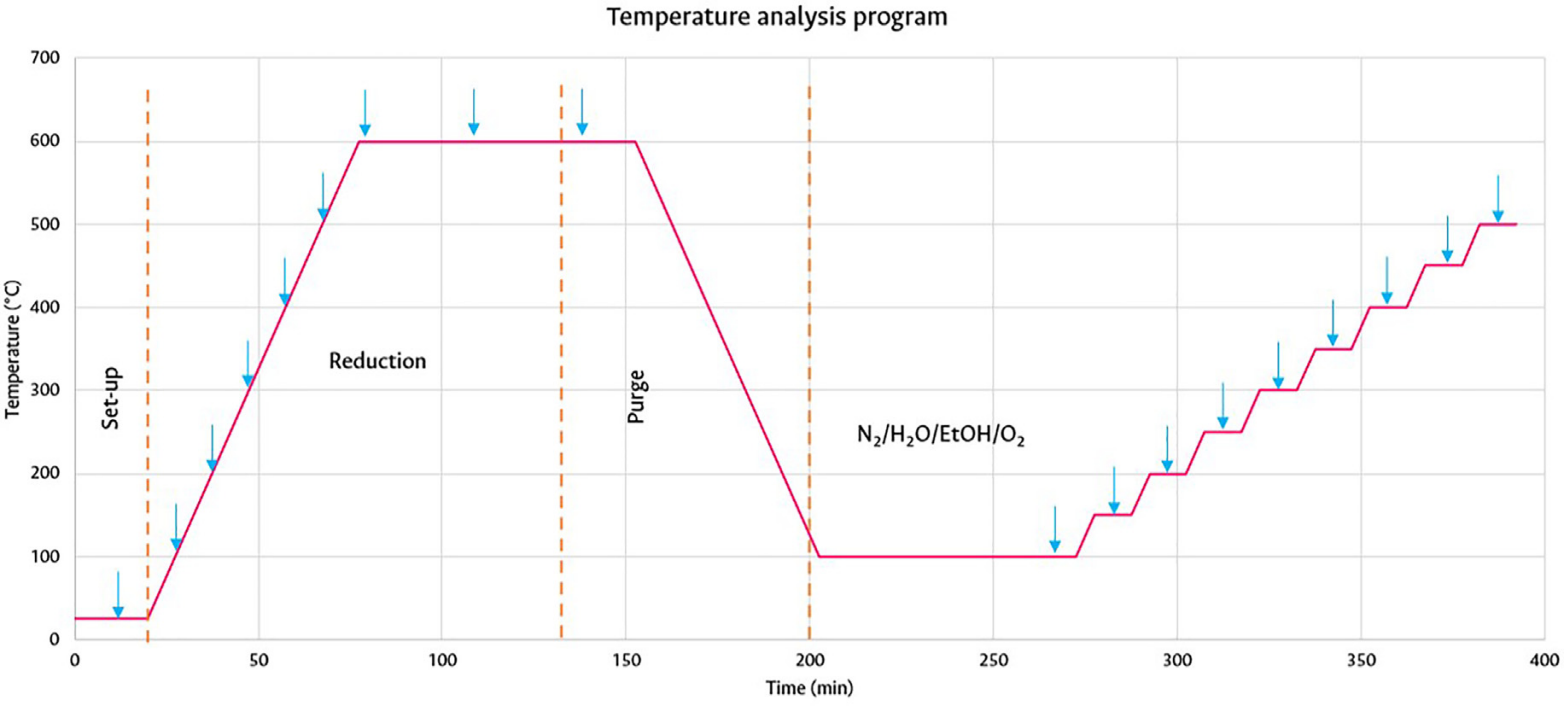
Methodological diagram for tests based on time.

Once the adsorption of ethanol was guaranteed, the evolution of the species based on temperature was evaluated. Fig. 8 illustrates the thermal method for the process at 100, 150, 200, 250, 300, 350, 400, 450 and 500 °C with a ramp of 10 °C·min^-1^, recording the spectra at each temperature (blue arrows).

**Fig. 9:**
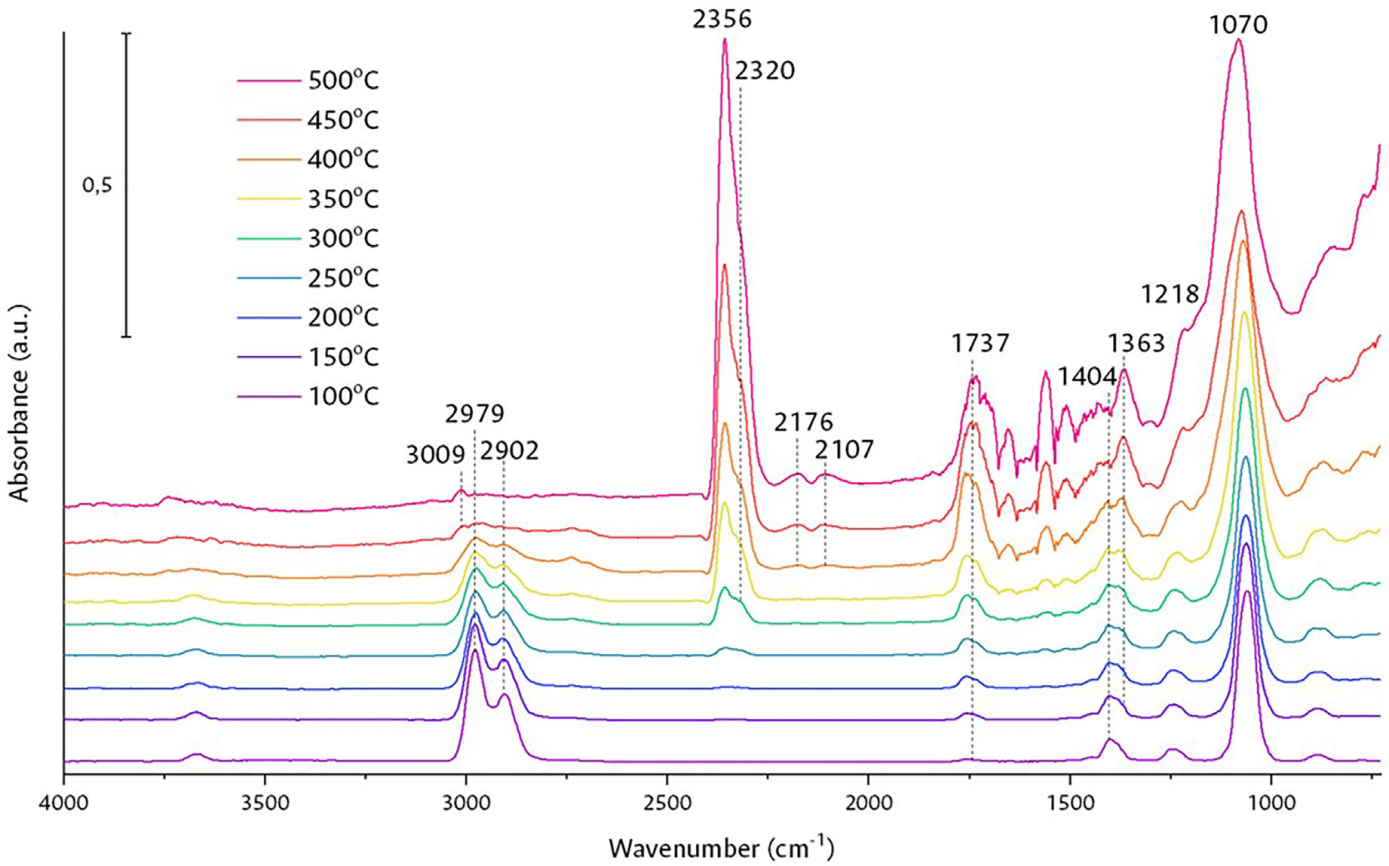
Target reaction with KBr under constant flow of the N_2_/H_2_O/EtOH/O_2_ mixture.

Fig. 9 illustrates the blank of the KBr diluent in the OSRE reaction, to evaluate its possible contribution during the process. The initial signals were maintained as the temperature increased: the ethoxy group signal did not disappear throughout the process, acetaldehyde signals appeared at 1737 cm^-1^ from 150 to 500 °C, at 300 °C of CO_2_, of the symmetric and asymmetric vibration at 2356 and 2320 cm^-1^, at 400 °C of CO at 2176 and 2107 cm^-1^, and of methane at 3009 cm^-1^ at 450 °C. Given the absence of a catalyst, these species as a whole indicate the thermal decomposition of ethanol [35,36] in the reaction chamber, evidencing that KBr has no effect on the reaction.

## Online measurement by MS

The simultaneous analysis by mass spectrometry (MS) allowed the detection of the species formed at the outlet of the gas mixture. According to the possible species that can be formed in the reaction established in literature reports and previous results of our research group [16], [17], [18], [19], [20], [21],^30^,31], Table 1 summarizes the corresponding signals in MS of each compound, revealing that not all the main signals (100% relative intensity) of the species are similar to their molecular weight due to the fractionation of the molecule in the ionization process.

**Table 1:** Assignment of the main signals in masses obtained from the NIST Mass Spectrometry Data center open database.

Compound (Molecular formula)	molecular weight (g·mol^-1^)	*m/z* (relative intensity%)					
		Species					
		1	2	3	4	5	6
Hydrogen (H_2_)	2.0	2.0 (100)	1.0 (2)	–	–	–	–
Methane (CH_4_)	16.0	16.0 (100)	15.0 (95)	14.0 (15)	13.0 (7)	12.0 (2)	–
Water (H_2_O)	18.0	18.0 (100)	17.0 (22)	16.0 (1)	–	–	–
Acetylene (C_2_H_2_)	26.0	26.0 (100)	25.0 (19)	13.0 (6)	24.0 (6)	27.0 (4)	–
Nitrogen (N_2_)	28.0	28.0 (100)	14.0 (13)	–	–	–	–
Carbon monoxide (CO)	28.0	28.0 (100)	12.0 (5)	16.0 (1)	–	–	–
Ethene (C_2_H_4_)	28.0	28.0 (100)	27.0 (64)	26.0 (61)	25.0 (12)	24.0 (4)	14.0 (4)
Ethane (C_2_H_6_)	30.0	28.0 (100)	27.0 (35)	30.0 (30)	26.0 (24)	29.0 (22)	15.0 (5)
Acetaldehyde (C_2_H_4_O)	44.0	29.0 (100)	44.0 (82)	43.0 (45)	15.0 (28)	42.0 (16)	14.0 (6)
Ethanol (C_2_H_5_OH)	46.0	31.0 (100)	45.0 (47)	29.0 (32)	27.0 (22)	46.0 (21)	26.0 (16)
Oxygen (O_2_)	32.0	32.0 (100)	16.0 (20)	–	–	–	–
Acetic acid (C_2_H_4_O_2_)	60.0	43.0 (100)	45.0 (92)	60.0 (75)	15.0 (18)	42.0 (14)	29.0 (10)
Acetone (C_3_H_6_O)	58.0	43.0 (100)	58.0 (23)	15.0 (13)	42.0 (10)	27.0 (8)	26.0 (5)
Carbon dioxide (CO_2_)	44.0	44.0 (100)	28.0 (11)	16.0 (11)	12.0 (9)	–	–

These species were loaded into a multiple detection program [15] over time in Quadera software. Most of these species do not present interference from their main signal, except for N_2_, CO, C_2_H_4_ and C_2_H_6,_ which present the same signal at *m/z* = 28.0. For these compounds it is difficult to show which product corresponds to said signal, however, a constant signal of N_2_ as carrier gas from the cleaning stage is taken as a baseline to attribute any change to the production of CO, C_2_H_4_ or C_2_H_6_ within the reaction.

To begin the experiment, the SEM and Faraday detectors were turned on in the Quadera software 15 min before the analysis, the Faraday detector works by measuring the change in electric current inside the Faraday cup at the moment when the ions collide with its wall and the SEM detector consists of a first ion-sensitive dynode, when the ions collide with it they produce secondary electrons, and the subsequent dynodes generate a cascade of electrons. These two detectors are the most widely used and are ideal for measuring gases and small molecules, achieving robust and easy detection of the generated ions. Then the inlet valve to the equipment was opened and the heaters of the inlet hose were turned on to avoid the condensation of liquid products such as water. Once the method with the created species is selected, the multiple detection of the species loaded in the program starts.

### Gas pulse test in MS

**Fig. 10:**
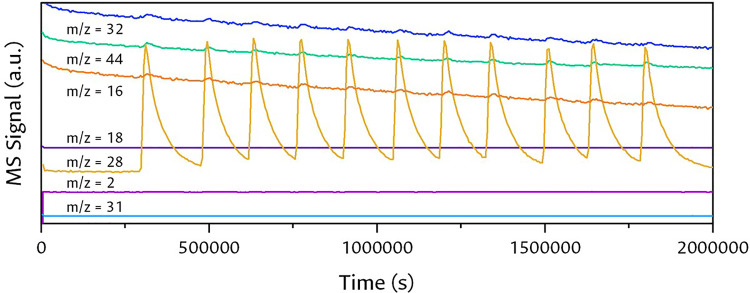
CO pulses through a bed of KBr arranged in the Harrick chamber at room temperature.

For the MS measurement test, a constant flow of N_2_ (30 ml·min^-1^) was introduced through the DRIFT cell loaded with KBr and the Quadera software program was immediately started, recording the constant signal of all species. Regular pulses of CO were then injected through the available loop in the 6-way valve. Fig. 10 reveals the change of the *m/z* = 28.0 signal generated by the entry of each CO pulse into the reaction system.

**Fig. 11:**
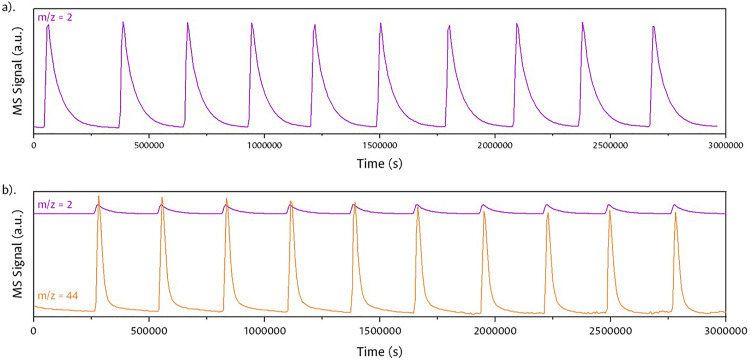
H_2_ injections and H_2__—_CO_2_ mixture.

The same test was performed with H_2_ and a mixture of H_2_ and CO_2_ (Fig. 11), detecting the signal of pure gases or mixtures of each pulse with a coefficient of variation (CV) of 0.07, without any other interfering signal. Through the one-way ANOVA statistical analysis of variance, the comparison of the injected pulses was carried out, finding that similar concentrations of the gases did not have significant variances (p) with a value < 0.05.

## End of test

Once the experiment is finished, the feed of the liquid mixture is suspended by turning off the HPLC pump and the air flow in the controller is stopped. Then, the lanes and reaction chamber are purged at the final temperature by passing N_2_ (30 mL·min^-1^) for 15 min. The resistances and the oven are then turned off, allowed to cool, the N_2_ flow is suspended and the chamber is disassembled to clean the reaction vessel. In the MS, the acquisition of the signals is stopped, the inlet valve is closed, and the SEM and Faraday detectors are turned off.

## Conclusions

This document reports in detail the experimental design for carrying out the study under operando DRIFT-MS conditions over time for the oxidative steam reforming of ethanol reaction (OSRE) which, to our knowledge, has not been reported under the mentioned conditions.

The experiments, carried out successfully, allowed to establish the conditions of analysis and preparation of the solid in an optimal way to achieve, on the one hand, signals with excellent resolution and, on the other hand, to demonstrate the importance of the use of KBr as a diluent, demonstrating the absence of any effect on the OSRE reaction.

The protocol allows us to spectroscopically observe the intermediate species generated on the surface, measure online the gaseous products generated and perform chemisorption on the catalyst at a given temperature. This will contribute to the understanding of: i) the dynamics on the catalyst surface, ii) the reaction mechanism and iii) the effect of solid properties on the catalytic process.

In this sense, the system DRIFT-MS established can not only allow monitoring of the OSRE reaction but also of others such as CO_2_ methanation and the WGS reaction that involve similar molecules and do not require temperatures above 600 °C.

## Declaration of Competing Interest

The authors declare that they have no known competing financial interests or personal relationships that could have appeared to influence the work reported in this paper.

## Data Availability

Data will be made available on request.
